# Physician and patient concordance of report of tobacco cessation intervention in primary care in India

**DOI:** 10.1186/s12889-015-1803-5

**Published:** 2015-05-02

**Authors:** Rajmohan Panda, Divya Persai, Sudhir Venkatesan, Jasjit S Ahluwalia

**Affiliations:** Public Health Foundation of India, New Delhi, India; Division of Epidemiology and Public Health, University of Nottingham, Nottingham, UK; Center for Health Equity, University of Minnesota, Minneapolis, MN USA

## Abstract

**Background:**

Tobacco cessation interventions by physicians hold promise in improving quit rates. The 5As intervention (‘*Ask’, ‘Advise’, ‘Assess’, ‘Assist’ and ‘Arrange’*) is an evidence-based approach for tobacco cessation. However, little is known about adherence with the tobacco cessation interventions in primary care in India. In the present study we assessed physicians’ adherence with the 5As intervention and explored physician and patient concordance on the report of 5As intervention for tobacco cessation.

**Methods:**

We used data from two cross-sectional surveys conducted in 12 districts of Andhra Pradesh and Gujarat in India. The surveys were administered simultaneously to both patients attending, and physicians working in health facilities providing primary care. Health facilities were selected by systematic random sampling and patients were recruited by simple random sampling. Common health facilities where both surveys were performed were identified, and individual patients were matched to their physicians through a unique matching code to obtain the two study samples.

**Results:**

Slight agreement was observed between the physician and patient responses on *‘Ask’* and *‘Arrange’* component of the 5As intervention. The ‘*Advise*’, ‘*Assess*’ and ‘*Assist*’ components showed low agreement. Slightly higher levels of agreement were seen on all components of the 5As, except ‘*Advise*’, for those patients who had made an attempt to quit.

**Conclusions:**

Our study suggests an urgent need for revising current strategies in order to strengthen the ‘*Advise*’, ‘*Assess*’, and ‘*Assist*’ interventions in tobacco cessation in primary care settings. Patient surveys should be used routinely in assessing fidelity and provider adherence for large scale behavioral health programs.

## Background

Tobacco use is one of the greatest causes of preventable deaths and disease in human history. According to the World Bank, four‐fifths of the world's 1.1 billion smokers live in low‐income or middle‐income countries [1]. As per GATS India data (2010) there are 275 million adult tobacco users (rural-216 million, urban-59 million) in India [2]. About one million Indians die from smoking alone each year, which is 15% of global death burden attributable to tobacco use [3]. To reduce the economic and health burden from tobacco use, effective tobacco cessation interventions are clearly needed.

Tobacco cessation interventions by healthcare providers hold promise in improving quit rates. Several studies have demonstrated that asking about smoking and offering advice about cessation increases quit rates [4,5]. However, little is known about the extent of adherence with the tobacco cessation interventions in primary care in India. A study by Panda et al. in 2011 among physicians in primary care facilities in India suggests that tobacco cessation interventions were not being offered in primary care clinics to any significant extent and patients were not benefiting from opportunistic counseling advice [6]. No simple empirically validated model captures the broad range of interventions across tobacco but the 5As construct provides a workable framework to report tobacco cessation interventions. The 5As include ‘*Asking’* all patients about tobacco use, ‘*Advising’* tobacco users to quit, ‘*Assessing’* tobacco users’ readiness to quit, ‘*Assisting’* patients in their quit attempts, and ‘*Arrange’* follow-up visits and counseling. Although the 5As approach is becoming more widely adopted as a strategy for behavior change counseling in tobacco cessation, practical and standardized assessments of 5As delivery are not widely available in the developing world and this is true also for India [7]. Accurate measures of providers’ delivery of tobacco cessation efforts during clinical practice are needed to monitor providers’ adherence to the 5As approach and to assess the impact of interventions [8]. Provider treatment of tobacco use can be measured by patient surveys, provider surveys, medical record reviews, and direct observation [9]. We assessed physicians’ adherence with the 5As intervention by conducting surveys simultaneously with both physicians and patients respectively. We also assessed the concordance on physicians’ and patients’ report of the 5As intervention by measuring extent of agreement between physicians and patients report on the 5As intervention. We further explored patients’ agreement on 5As intervention in relation to their quitting behavior. The study was conducted in selected health facilities providing primary care.

## Methods

### Study settings and design

We used data from two cross-sectional surveys conducted in 12 districts of Andhra Pradesh and Gujarat in India from June to August 2013. The two surveys were administered simultaneously among both patients as well as physicians working in health facilities providing primary care.

Health facilities providing primary care in India include Primary Health Centers (PHC), Community Health Centers (CHC), and sub-district and district hospitals. Each primary and community health center caters to the population of 20,000-30,000 and 80,000-120,000 respectively hospitals [10]. Primary healthcare in India is provided by a variety of healthcare providers including doctors trained in medicine, and practitioners trained in the indigenous systems of medicine (Ayurveda, Unani, Siddha, and Homeopathic medicine-AYUSH) [11].

### Study group

#### Physicians’ survey

The health facilities were chosen using systematic random sampling. All the health facilities providing primary care in the district were listed. The first health facility was selected at random and then every fifth health facility was selected for inclusion in the sample. All consenting physicians practicing at the selected facilities were surveyed for inclusion into the study. The physicians’ survey included a) Background characteristics, b) Practices in tobacco cessation c) Knowledge of physicians in tobacco cessation interventions, d) Attitude towards tobacco cessation. Responses to the 5As interventions were coded as either ‘yes’ or ‘no’. Physicians who self-reported that they ‘*Ask’* patients about tobacco use and who counseled about tobacco cessation were considered to have fulfilled the criteria for ‘*Ask’* and ‘*Advise’* respectively. ‘*Assist’* was measured by any of the following questions: During the past 12 months, did you suggest nicotine replacement therapy such as a patch or gum? or during the past 12 months, did you perform one time counseling to your patients to help them quit? or have you ever given any printed material to the patient? Information on follow-ups to higher centers for cessation determined the ‘*Arrange’* component of the 5As intervention.

A situational analysis and literature review was conducted which help developed the questionnaire. Prior formative research was done to determine themes of the questionnaire. The questionnaire was administered by trained interviewers hired from a survey agency. The questionnaire was validated and pilot tested and changes were made. The interviews were conducted in local language. The interviewers established good rapport with the respondents before administrating the questionnaire. To reduce the social desirability bias, respondents’ names were kept anonymous and confidentiality was maintained.

#### Patients’ survey

Patients were approached immediately following the patient-physician interaction. The study participants were recruited through simple random sampling by selecting every third patient who registered to see the health service provider on each consulting day during the study period excluding weekends and public holidays. The participant eligibility was determined on the basis of whether the respondent was adult (more than 18 years), sought services from health service providers, and consumed tobacco in some or the other form. The questionnaire was administered by trained interviewers hired from a survey agency. The questionnaire was validated and pilot tested and changes were made. Data collection was done at suitable places near to the vicinity of health facilities away from the consultation rooms. Critically ill patients, those younger than 18 years, and those who did not give consent were excluded from the study. The patients’ survey included a) Participant eligibility, b) Socio-demographic information, c) Tobacco use information, d) Tobacco counseling practices by physicians working in health facilities providing primary health care and e) Motivation to quit. Data were collected on patient receipt of the 5As through specific questions. The patient reported ‘*Ask’* variable was measured with the question ‘Have you been ‘*Asked’* about your tobacco consumption habit during today’s visit?’ ‘*Advis*e’ was assessed with the question ‘Did your physician advise you to quit tobacco?’ Physician provision of ‘*Assess’* were measured with any of the following questions: Did your physician inform you about different medicines for quitting? or Did your physician suggest ways to quit tobacco use? or Did your physician provide you any printed material?

Survey questions used to capture patients’ and physicians’ responses on the 5As interventions are summarized in Table 1.

**Table 1:** **Survey questions used to capture data on the 5As**

**5As**	**Patients’ survey**	**Physicians’ survey**
**Ask**	Have you been asked about your tobacco consumption habit during today’s visit?	Do you take the history of tobacco usage of the patients who come to you?
**Advise**	Did your physician advise you to quit tobacco?	Do you tell people who come to you to quit tobacco use?
**Assess**	Did your physician ask for your willingness to quit?	Do you ask whether patient is willing to quit tobacco?
**Assist**	Did your physician inform you about different medicines for quitting?	During the past 12 months, did you suggest nicotine replacement therapy such as a patch or gum?
	Did your physician suggest ways to quit tobacco use?	During the past 12 months, did you perform one time counselling to your patients to help them quit?
	Did your physician provide you any printed material (take away material)?	Have you ever given any printed material (take away material) to the patient?
**Arrange**	Did your physician inform you about the further follow up at higher centres?	During the past 12 months, did you arrange for counselling with follow-ups?
	Did your physician tell you when to return for follow-up counselling?	

### Data analysis

Common health facilities where both surveys were performed were identified, and individual patients were matched to their physicians through a unique matching code to obtain the two study samples. The data collection forms for these assessments compared a common set of interventions based on the structure of the 5A algorithm and agreement between the patient and physician responses was assessed.

The main predictors of interest for the present analysis were patient and physician self-reported 5As intervention in tobacco cessation. Percentage agreement for each component of the 5As was calculated to assess agreement between the physicians’ survey and the patients’ survey. The analyses were performed using Stata 12.0 (StataCorp, 2011. Stata Statistical Software: Release 12.).

The study was approved by the Public Health Foundation of India, Institutional Ethical Committee (IEC 65/60). Informed consent was taken from both physicians and patients.

## Results

### Background characteristics

#### Physicians’ characteristics

A total of 345 physicians were interviewed. Out of these 345 physicians, there were 293 physicians who were practicing at the health facilities where physician and patient survey were conducted. The majority of physicians were male (69%) and the survey response rate was 95% (345/384). At the health facilities, physicians had multiple encounters with the patients. The average number of patients seen by physicians per day in both the states was 80. The surveyed health facilities consisted of Primary Health Centers (58%), Community Health Centers (40%) and District Hospitals (2%). About 242 (82.6%) of physicians had a medical degree, 17 were dental graduates (5.8%), 33 had Ayurveda, Unani, Siddha, and Homeopathic medicine (AYUSH) qualification (11.3%) and 1 (0.3%) had a government approved professional certificate. Background characteristics of physicians are summarized in Table 2.

**Table 2:** **Background characteristics of physicians (n = 293)**

**Characteristic**	**n (%)**
**Age median (IQR) in years**	33 (29 to 41)
**Sex**	
Female	91 (31)
Male	202 (69)
**Place of Work**	
District Government Hospital	1 (0.3)
CHC	118 (40)
PHC	171 (58.3)
Urban Health Centre	3 (1)
**Highest qualification**	
MBBS (Bachelor of medicine and Bachelor of surgery)	236 (80.5)
MDS (Masters in Dental Surgery)	2 (0.7)
BDS (Bachelor of Dental Surgery)	15 (5)
BAMS (Bachelor of Ayurvedic Medicine and Surgery)	20 (7)
BUMS (Bachelor of Unani Medicine and Surgery	1 (0.3)
BHMS (Bachelor of Homeopathic Medicine and Surgery)	5 (1.7)
BEMS (Bachelor of Electropathic Medicine and Surgery)	7 (2.4)
MD (Doctor of Medicine)	2 (0.7)
MS (Master of Surgery)	4 (1.4)
Other Professional Certificate course by Government	1 (0.3)

#### Patients’ characteristics

A total of 867 patients who were tobacco users and made outpatient visit to the health facility providing primary care were recruited for the study. The response rate of the patient survey was 97%. About 46% of patients were smokers, 42% were smokeless tobacco users and 12% of them were dual tobacco users. About 68% of the patients had not made an attempt to quit tobacco in past 12 months, with the remaining having made at least one attempt to quit. Table 3 summarizes the background characteristics of patients.

**Table 3:** **Background characteristics of patients**

**Form of tobacco**	
Smoked tobacco (%)	402 (46)
Smokeless tobacco (%)	360 (42)
Both (%)	105 (12)
**Presenting Illness**	
General Ailments (%)	474 (55)
Respiratory complaints (%)	344 (40)
Others (%)	49 (5)
**Number of visits in the past 12 months**	
1 or 2 times (%)	391 (45)
3 to 5 times (%)	377 (44)
6 times or more (%)	99 (11)

### Physicians’ report of 5As intervention in tobacco cessation

Over 90% of physicians self-reported that they ‘*Ask*’ patients about tobacco use, ‘*Assess*’ readiness to quit, ‘*Assist*’ in quitting and ‘*Arrange*’ follow-up visits. Only 44% of physicians said that they *‘Advise’* patients to quit tobacco.

### Patients’ report of 5As intervention in tobacco cessation

More than two-third of the patients reported that they were ‘*Asked’* about tobacco usage (77%) by the physicians. About half of patients reported they were ‘*Advised’* to quit (51%) and ‘*Assessed’* for their interest in quitting (48%), about a third of patients were offered a follow-up (*Arranged*) contact (38%).

### Concordance between physicians and patients on each component of the 5As intervention in tobacco cessation

**Figure 1:**
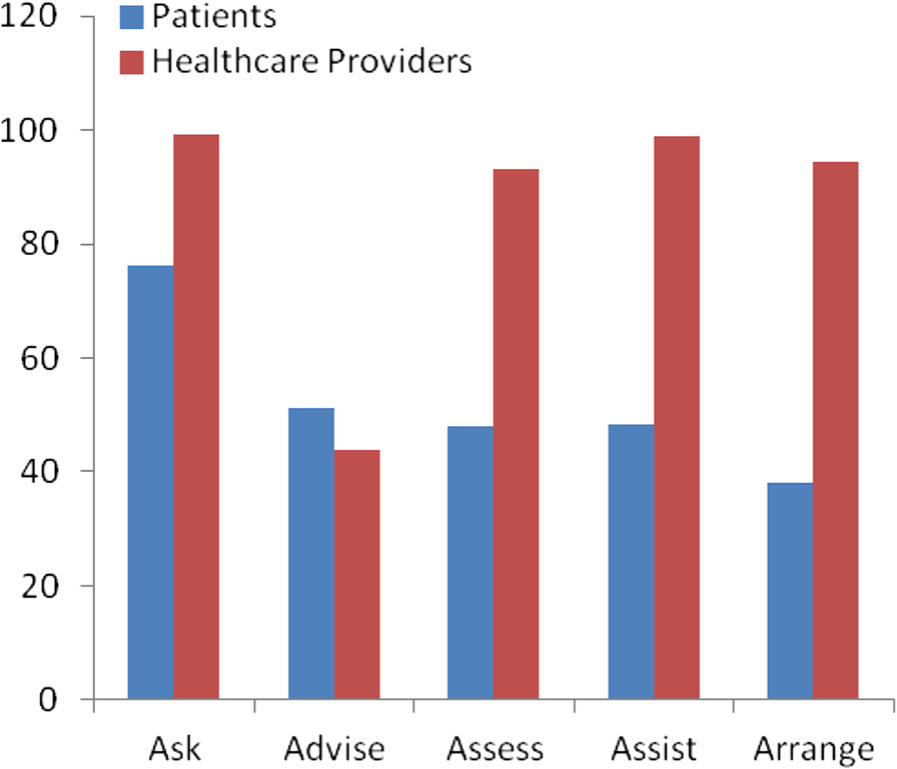
Proportion of patients and physicians reporting each of the 5As.

Proportions of physicians’ and patients’ report of the 5As intervention are presented in Table 4. Figure 1 depicts this data graphically. Physicians were seen to have reported consistently higher levels of the 5As than patients reported receiving them except the ‘*Advise*’ component which was reported at a slightly higher frequency by patients (51%) when compared to physician (43.7%). *‘Ask’* and ‘*Advise’* components of the 5As showed the least difference in percentages reported between the groups with ‘*Assess’, ‘Assist’, and ‘Arrange’* showing increasingly higher percentage differences.

**Table 4:** **Proportions reporting each component of the 5As**

	**Patients (%)**	**Physician (%)**	**% Difference**
**Ask**	661 (76.2)	291 (99.3)	23.1
**Advise**	443 (51.1)	128 (43.7)	7.4
**Assess**	417 (48.1)	273 (93.2)	45.1
**Assist**	419 (48.3)	290 (99)	50.7
**Arrange**	330 (38.1)	277 (94.5)	56.4

Percentage agreement between physicians and patients was the highest for *‘Ask’* (76.36%). The remaining three components of the 5As showed low agreement between the two surveys (Table 5). Our findings indicate that percentage agreement was higher among patients who were smokeless tobacco users as compared to those who were smokers across all the 5As interventions. When the patient population was split into those who had made at least one quit attempt in the past twelve months and those who had not, slightly higher levels of agreement were seen in all components of the 5As, except *‘Advise’*, for those patients who had made an attempt to quit. Conversely, amongst those who had not made an attempt to quit over the past twelve months, slightly lower levels of agreement were seen for all components, except *‘Advise’*.

**Table 5:** **Agreement between patients and physicians on each component of the 5As**

	**Overall n = 867**	**Number of quit attempts made (past 12 months)**			**Form of tobacco used**	
		**At least 1 quit attempt**	**No quit attempts**	**Smoked tobacco**	**Smokeless tobacco**	**Both forms**
	**% agreement**	**% agreement**	**% agreement**	**% agreement**	**% agreement**	**% agreement**
**Ask**	76.4	85.4	72.1	73.9	78.6	78.1
**Advise**	45	34.3	50.1	43.8	44.7	50.5
**Assess**	47.1	56.1	42.8	41.3	52.2	51.4
**Assist**	48.8	53.6	46.5	39.1	56.1	61
**Arrange**	41.4	43.9	40.2	34.3	47.5	47.6

## Discussion

The 5As intervention is an evidence-based approach for tobacco cessation and is feasible to apply in primary care. This study describes adherence with the 5As intervention for tobacco cessation and concordance between patient**-**provider delivery and receipt of 5As interventions respectively. We captured both patients’ and physicians’ responses simultaneously without a lag period. Globally, patients’ view on quality of behavioral interventions has been captured in previous studies [12,13] however, no studies have assessed physician and patient report of the 5As in tobacco cessation in India.

The findings of the current study are consistent with the findings of previous work [14,15] in which the majority of physicians self-report that they ‘*Asked’* patients about tobacco use. Our finding related to asking patients about tobacco use is also in agreement with the study by Conroy et.al. [16] where 76% of the patients reported that they were asked for tobacco use during their visit to physicians. However, on examining physicians and patients responses we found that there is slight agreement between physicians’ and patients’ responses regarding the *‘Ask’* component of 5As intervention. The high percentage reported on *‘Asking’* component by patients is promising as asking for tobacco use is very often the first step towards a more comprehensive cessation intervention.

Our data suggest that most physicians do not ‘*Advise’* patients to quit. Similar findings were observed in GATS, India (2010) data [2], studies by Thankappan et.al. [17], and Mas et.al. [18], which also found that less than 50% of physicians are routinely advising patients to quit tobacco. When we assessed agreement between physicians and patients report, we found low agreement on ‘*Advise’* component of 5As intervention as has also been reported by Pollak et.al, in 2002 [19]. When patients attend primary health facilities, an enquiry about tobacco exposure by a physician and brief advice to quit can increase the rates of tobacco cessation [20]. Unfortunately, these opportunities were largely missed by physicians in our study.

A recent meta-analysis highlights the effectiveness of *‘Assess’* intervention and suggests that prior assessment of willingness to quit excludes many tobacco users who would have taken up the offer of assistance if offered directly [21]. However, clinical practice guidelines recommend assessment of willingness as an important step which further provides a roadmap for tobacco cessation treatment [10]. Our findings indicate that though a majority of physicians self-reported that they *‘Assess’* patient willingness to quit, only a few patients reported being *‘Assessed’* by physicians. Low agreement on ‘*Assess’* intervention between patients’ and physicians’ report was observed. Although it is possible that patients underestimated physician assessment, the fact that they did not recall a physicians’ assessment strategy is clinically important and suggests the need for more intensive interventions in the primary care setting.

In contrast to physicians’ self-reported practices, patients in our study mentioned that only a few physicians ‘*Assisted’* them with their quit attempts. Similar findings were observed in studies conducted in other settings [22,23]. Our findings also indicate low agreement between physicians and patients on the ‘*Assist’* component of 5As. This is a cause for concern as it suggests that physicians are not offering adequate support to help patients quit tobacco despite strong recommendations by national guidelines which give emphasis to assist patients in quitting tobacco [10].

In our study, the majority of physicians self-reported that they ‘*Arrange*’ follow-up visits for the patients. However, patient surveys reveal contrasting findings. We found slight agreement on ‘*Arranging’* for follow-up visits and this finding is similar to a study reported by Omole et.al.in South Africa in 2010 [23]. The slight agreement on follow-up support is promising and underscores the need for a pragmatic approach incorporating checklists into system reminders to prompt physicians to provide information on follow-up counseling sessions and support [9].

Similar to the findings of our study, studies conducted in other South**-**Asian countries reported discordance between rates of ‘*Advise’, ‘Assess’, ‘Assist’* between physicians and patients [24,25]. We reason that this discordance could be because patients often underestimate physicians’ interventions. The concern of patients about their personal medical problem may have affected their responses [26].

While many studies report on the issues of concordance, there are few which have examined the relationship of agreement to quitting behavior. We explored the association of agreement on 5As intervention with patients’ quit attempts. Our findings are similar to the findings reported by Quinn et al. who suggests that patient who made a quit attempt had higher agreement on receipt of 5As as compared to those patients who did not attempt to quit [26].

Our findings, although insightful, need to be interpreted cautiously as the patient survey was conducted in a subsample of health facilities providing primary care, and thus may not be representative of the overall health care experience in primary care settings. The patients’ responses were captured at the time of their visit to the health facility. However, the questionnaire captured physicians’ responses and 5As interventions over a period of 12 months. Thus, physicians were more likely to overestimate their practices in the present study. Further, these results are based on reports from patients and do not reflect the notations from the medical record. Ideally, an audit of the physicians’ records would better validate findings from the surveys. However, considering that medical records of physicians are not currently maintained in India as in other developing countries, patient interviews are likely to be the best available evidence on the 5As interventions of the physicians.

## Conclusions

In conclusion, concordance between physician and patient self-report is slightly higher for ‘*Ask’* and *‘Arrange’* and low for ‘*Advise’, ‘Assess’*, and *‘Assist’* interventions. Our study suggests an urgent need for revising current strategies in order to strengthen the ‘*Advise’, ‘Assess’*, and *‘Assist’* interventions in tobacco cessation in primary care settings. This study helped developed what we believe is the first comparison of behavioral health interventions (5As) in India by using two standard surveys capturing both physician as well as patient responses at the same time.

We propose that patient surveys such as ours should be used routinely in assessing fidelity and provider adherence for large scale behavioral health programs especially in the areas of non-communicable disease program. Further research is needed in order to determine the true rate of tobacco cessation intervention in primary care settings, assess barriers towards provision of tobacco cessation services as well as to verify trends in patient and physician reports in India and other developing countries.
